# The outcome of patients with inflammatory bowel disease–associated colorectal cancer is not worse than that of patients with sporadic colorectal cancer–a matched-pair analysis of survival

**DOI:** 10.1007/s00384-021-04072-9

**Published:** 2021-12-04

**Authors:** Francesco Vitali, Axel Wein, Timo Rath, Markus Eckstein, Clemens Neufert, Jürgen Siebler, Raja Atreya, Arndt Hartmann, Werner Hohenberger, Klaus Weber, Markus Friedrich Neurath, Robert Grützmann, Susanne Merkel

**Affiliations:** 1 Department of Internal Medicine 1, Friedrich-Alexander-University Erlangen-Nuremberg, Ulmenweg 18, 91054 Erlangen, Germany; 2 Institute of Pathology, Friedrich-Alexander-University Erlangen-Nuremberg, Ulmenweg 18, 91054 Erlangen, Germany; 3 Department of General and Abdominal Surgery, Friedrich-Alexander-University Erlangen-Nuremberg, Ulmenweg 18, 91054 Erlangen, Germany

**Keywords:** Inflammatory bowel disease, Colorectal cancer, Ulcerative colitis, Crohn’s disease

## Abstract

**Purpose:**

Patients with inflammatory bowel disease (IBD) have an increased risk for colorectal cancer (CRC). In IBD patients, cancer is often diagnosed in advanced stages and conflicting data on survival compared to sporadic CRC have been reported. The aim of this study was to directly compare clinical characteristics and prognosis of patients with IBD-CRC and sporadic CRC.

**Methods:**

The clinical and pathological data of 63 patients with IBD-CRC and 3710 patients with sporadic CRC treated at the University Hospital of Erlangen between 1995 and 2015 were compared. Forty-seven M0 patients with IBD were matched with sporadic CRC patients after curative resection (R0) according to tumor localization, stage, sex, and year of treatment. Overall and disease-free survival were compared.

**Results:**

Sixty-three patients presented IBD-CRC. Fifty were affected with ulcerative colitis (UC) and 13 with Crohn’s disease (CD). CRC was diagnosed within 1.45 years since last endoscopic surveillance. Twelve patients (19%) had a diagnosis of primary sclerosing cholangitis. In matched analysis, IBD patients were diagnosed with CRC at younger age compared to sporadic CRC and were more likely to have right-sided CRC (40% versus 23.3%) and rare histological subtypes (19% versus 9.2%). No differences in 5-year overall (78.7 versus 80.9 months) and 5-year disease-free survival (74.5 versus 70.2 months) were noted.

**Conclusion:**

IBD-CRC patients were younger and more frequently had right-sided carcinomas compared to sporadic CRC. CRC in IBD patients did not show survival difference compared to matched-pair sporadic CRC patients without distant metastases after curative resection. Surveillance might be important for early detection of CRC in IBD patients.

## Introduction

Inflammatory bowel disease (IBD) is an established risk factor for the development of colorectal cancer (CRC) [1, 2]. Enhanced risk of CRC in IBD is associated with the severity and duration of inflammation, extent of disease (particularly in cases of pancolitis), smoking, family history of CRC, or the presence of primary sclerosing cholangitis [2–7]**.** As shown in several reports including prospective trials, the risk of colorectal cancer in UC is related to the cumulative inflammatory burden (CIB) as a feature of active endoscopic and histological inflammation [8–12].

Although an early meta-analysis of 116 studies has estimated a cumulative risk in ulcerative colitis of 18% after 30 years of disease duration [4], more recent studies noted a lower cancer incidence. As such, the excess CRC risk of 2.4 [13] has been estimated. For those patients with colonic Crohn’s disease, the CRC risk has been estimated at a standardized incidence ratio (SIR) of 1.9 [14]. The patchy distribution of CD could explain the lower CRC risk compared to patients with UC. However, the risk of CRC in long-standing Crohn’s colitis patients is comparable to those with UC, since a meta-analysis of data from population-based studies found a pooled SIR for CRC of 1.7 (95% confidence interval, 1.2–2.2) in all patients with IBD [15]. The prospective *CESAME* Cohort confirmed an increased SIR of 7.0 for CRC in patients with long-standing extensive colitis [8]. According to a national nationwide study, patients with IBD who were diagnosed with CRC were younger than sporadic CRC counterparts [16, 17]. They were also more likely to have multiple neoplastic lesions and had higher proportions of superficial-type lesions and invasive-type lesions at histology, as well as mucinous or signet ring cell histotypes [17].

Among IBD patients, cancer may develop as a result of chronic inflammation, leading to dysplasia and then to carcinoma [18, 19]. Based on this mechanism of colitis-associated carcinogenesis, precancerous lesions in IBD are frequently heterogeneous and both histologically and molecularly different from sporadic colorectal carcinogenesis [20]. Commonly referred to as “field effect “ or “field cancerization,” inflammatory changes lead to a positive selection of heterogeneous genetic mutations resulting in a multitude of variants of colitis-associated precancerous lesions in which molecular alterations develop in normal-appearing tissue and expand into premalignant patches with the potential to progress to dysplasia and carcinoma [21, 22]. At the same time, most of the molecular dysregulations occurring in sporadic colorectal carcinogenesis are also detected in IBD-associated CRC; however, marked differences in both its timing and prevalence have been noted. Recent studies in surgical specimen revealed frequent early loss or overexpression of the oncogene p53, early KRAS mutation, infrequent BRAF mutation [20], presence of aneuploidy [23], MGMT (O6-alkylguanine DNA alkyltransferase) loss [24], and MMR deficiency in about 10% of cases [25, 26]. DNA methylation may also account for differences in presentation and outcomes between inflammatory bowel disease–associated carcinomas and sporadic CRC [27]. Molecular pathophysiological mechanisms of colitis-associated cancer are unclear; however, in a mouse model, the epiregulin/ERK (extracellular signal-regulated kinase) pathway appears to be particularly relevant for inflammation-associated colorectal carcinomas because mucosal inflammation results in marked production of potent epiregulin stimulators such as TNF-alpha and LPS (lipopolysaccharide-microbial proteins and pro-inflammatory molecules) [28].

Although many data on the risk factors for developing CRC in IBD patients and about strategies for prevention and early detection of CRC in IBD patients are available, knowledge about the presentation, treatment, and outcomes of CRC in IBD are scarce to date. Overall survival after CRC diagnosis in IBD patients is primarily determined by age, comorbidities, and cancer stage at diagnosis [1]. In the literature, IBD patients are found to be affected with CRC at younger age, are more often diagnosed in advanced stage, and have significantly decreased survival compared to sporadic CRC [29, 30]. However, Ali et al. could demonstrate in a propensity score matching analysis that the survival times of CRC patients with and without IBD were not significantly different [16].

Based on this, we set off to directly compare clinical characteristics and prognosis of patients with IBD-associated CRC and sporadic carcinogenesis.

## Patients and methods

The study was designed as a retrospective analysis of a prospectively maintained data from the Erlangen Registry for Colorectal Carcinoma (ERCRC), Department of Surgery, Erlangen, Germany. The study was based on data from 1 January 1995 to 31 December 2015. General epidemiologic data, clinical findings, treatment, and follow-up data were collected prospectively. When performed, data about molecular pathology findings inclusive analysis for microsatellite instability were recorded. The Eastern Cooperative Oncology Group (ECOG) performance status (PS) was recorded before resection of the primary tumor. Follow-up data were collected either at the university hospital or from review of written correspondence with the patients’ primary care physicians and specialists in gastroenterology and oncology. This study was approved by the Clinical Ethics Committee of the Friedrich-Alexander-Universität Erlangen-Nürnberg (FAU) (file number: 132_20 Bc).

Inclusion criteria for the study were as follows: age >  = 18 years, patients with inflammatory bowel disease–associated (Crohn’s disease, ulcerative colitis) colorectal carcinomas and sporadic carcinomas who underwent radical surgery with TME or CME [31, 32] between 1995 and 2015. Exclusion criteria were appendix carcinoma and for the matched-pair analysis metastatic CRC (stage IV) and non-curative resection.

Carcinomas were classified according to the UICC/AJCC 8th edition [33, 34]. Tumor classification of histomorphology followed the rules of the WHO [35]. Localization within the colorectum was subdivided into right colon (from the caecum to the proximal two-thirds of the transverse colon), left colon (from the distal third of the transverse colon to the sigmoid colon), and rectum.

The Clavien-Dindo classification was used to categorize postoperative complications [36].

### Matching

Patients with IBD-associated CRC were matched with patients with sporadic CRC according to the following factors: pathological T and N categories (UICC stage I–III), sex, ECOG performance status, and localization of the neoplasm. Because sporadic colorectal cancer without known genetic predisposition in younger age is a rarity, matching for age 1:1 at time of diagnosis was impossible, so the best possible matching individual was included. The matched-pair analysis also considered the year of treatment ± 5 years. A matching partner was found for 47 of the 48 IBD patients (M0, R0).

### Statistical analysis

Categorical data were compared using the chi-squared test, and when appropriate (≤ 5 patients within a group), the Fisher exact test was used. For comparison of continuous data, the Mann–Whitney *U* test was applied. The survival analysis was performed using the Kaplan–Meier estimation with the date of initiation of tumor-related therapy (resection or neoadjuvant therapy) as starting point. Overall and disease-free survival curves for both groups were compared using the log-rank test. For disease-free survival curves, an event was defined as the first occurrence of locoregional recurrence, distant metastases, or death from any cause. A *p*-value less than 0.05 was considered significant. Analyses were performed using the IBM® SPSS® Statistics 24.0 software package (Armonk, NY, USA).

## Results

We identified a total of 63 patients with IBD-related CRC (Table 1). Fifty patients were affected with UC and 13 with CD.

**Table 1:** Patient characteristics of 63 patients with IBD-associated colorectal carcinoma

Number of total: 63 pts	Ulcerative colitis (*n* = 50)	Crohn disease (*n* = 13)	
Sex (M/F)	29/21	6/7	*P* = 0.444
Alter	49.0 (± 16.7)	43.2 (± 9.9)	*P* = 0.132
BMI	27.9 ± 11	22.6 ± 3.6	*P* = 0.173
Smoking (> 5 Py)	1 (0.2%)	4 (30%)	*P* = *0.004*
Years of disease	18.5 (± 9.9)	19.0 (± 5.9)	*P* = 0.812
Surveillance colonoscopy performed	42 (84%)	13 (100%)	*P* = 0.333
Presence of DALM	30 (60%)	5 (38.4%)	*P* = 0.257
Cortison dependance	14 (28%)	6 (46%)	*P* = 0.245
Immunotherapy	1 (0.2%)	1 (7%)	*P* = 0.298
5-ASA therapy	33 (66%)	8 (61.5%)	*P* = 0.400
Immunosuppression	9 (18%)	5 (38%)	*P* = 0.153
MSI	5 (1%)	4 (30%)	*P* = 0.072
Active disease	49 (98%)	13 (100%)	*P* = 0.251
PSC	8 (1.6%)	4 (30%)	*P* = 0.486
Chemotherapy	24 (48%)	11 (84.6%)	*P* = 0.124
Immunotherapy	1 (0.2%)	1 (7.7%)	*P* = 0.291
Double colorectal cancer	5 (1%)	1 (7.7%)	*P* = 0.800
Stenosis	31 (62%)	7 (53.8%)	*P* = 0.591
Synchronous inflammation and cancer	37 (74%)	10 (77%)	*P* = 0.842
Colitis extension			*P* < *0.001*
- Rectum - Sigmoid colon - Left-sided - Pancolitis - Ileocoeal	2 5 6 37 0	0 2 1 7 3	

*BMI* body mass index, *MSI* microsatellite instability, *PSC* primary sclerosing cholangitis, *DALM* dysplasia-associated lesion or mass, *5-ASA* 5 aminosalicilate, *Py* pack years

Overall, IBD patients were diagnosed with CRC younger than patients with sporadic CRC (about 20 years earlier; Table 2). IBD patients presented more frequently with a right-sided CRC (40% versus 23.3%) and presented more often rare histological subtypes of CRC (19% versus 9.2%) (Table 2; *n* = 3773).

**Table 2:** Patient characteristics of IBD patients with colorectal carcinoma versus sporadic colorectal carcinomas, Erlangen Registry of ColoRectal Carcinomas (ERCRC), *n* = 3773

		IBD-associated CRC (*n* = 63)	Sporadic CRC (*n* = 3710)	*p*-value
		*n* (%)	*n* (%)	
Age	Median (range) (years)	45 (22–80)	66 (18–99)	< 0.001
Sex	Male	35 (56)	2315 (62.4)	0.266
	Female	28 (44)	1395 (37.6)	
ECOG*	ECOG 0–1	49 (85)	2882 (84.8)	0.953
	ECOG 2–4	9 (16)	518 (15.2)	
ASA**	ASA 1–2	49 (86)	2422 (75.2)	0.062
	ASA 3–4	8 (14)	797 (21.5)	
Tumor site	Right colon	25 (40)	863 (23.3)	0.008
	Left colon	12 (19)	1060 (28.6)	
	Rectum	26 (41)	1787 (48.2)	
Emergency surgery	Yes	5 (8)	246 (6.6)	0.680
	No	58 (92)	3464 (93.4)	
Surgery	TME/CME	36 (57)	3701 (99.8)	< 0.001
	(Procto)colectomy	27 (43)	9 (0.2)	
Histological type	Adenocarcinoma	51 (81)	3370 (90.8)	0.007
	Other types***	12 (19)	340 (9.2)	
Stage (UICC)	Stage I	16 (26)	748 (20.2)	0.334
	Stage II	17 (27)	790 (21.3)	
	Stage III	13 (21)	717 (19.3)	
	Stage IV	10 (16)	585 (15.8)	
	Stage y0	0	98 (2.6)	
	Stage yI	0	191 (5.2)	
	Stage yII	2 (3)	196 (5.3)	
	Stage yIII	1 (2)	202 (5.4)	
	Stage yIV	3 (5)	181 (4.9)	
Distant metastases	M0	50 (79)	2946 (79.4)	0.993
	M1	13 (21)	764 (20.6)	
R classification	R0	51 (81)	3097 (83.5)	0.345
	R1	2 (3)	53 (1.4)	
	R2	8 (13)	515 (13.9)	
	RX	2 (3)	45 (1.2)	
Multimodal treatment	Yes	31 (49)	1826 (50.8)	0.998
	No	32 (51)	1884 (49.2)	

*IBD* inflammatory bowel disease, *CRC* colorectal carcinoma, *ASA* American Society of Anesthesiologists Classification, *TME* total mesorectal excision, *CME* complete mesorectal excision; *ECOG performance status in 315 patients unknown; **ASA missing in 497 patients; ***undifferentiated carcinoma, mucinous adenocarcinoma, signet ring cell carcinoma, medullary carcinoma, adenosquamous carcinoma. RX usually due to complete radio frequency ablation of liver metastases

Patients with CD were more often smokers than patients with UC. UC patients with IBD-CRC presented more frequently with a pancolitis than with localized left-sided colitis or proctitis. Sex, age at onset of CRC, duration of IBD, the presence of primary sclerosing cholangitis, immunosuppression, and rate of corticosteroid medication dependence were not statistically different between UC and CD patients. Sixty-three patients (98.4%) presented with endoscopically active disease activity: one patient presented with endoscopic remission, 32 (50%) had moderate endoscopic activity, and 30 (48%) had severe endoscopic activity. The majority of the patients (*n* = 56; 88.9%) underwent complete surveillance colonoscopy. In these patients, CRC was diagnosed within 1.45 years (range 1–7, SD 1.25 years) since the last surveillance colonoscopy. One patient with UC was found to have CRC in a colon without inflammation (quiescent IBD). In this patient, who also had PSC, random biopsies removed foci of adenocarcinoma on a DALM (dysplasia-associated lesion or mass) and no other foci of colorectal adenocarcinoma were found in the surgical specimen.

Forty-seven (74.6%) patients had inflammatory activity synchronous with cancer; however, 16 patients (25.4%) presented cancer even in the absence of overt endoscopic mucosal inflammation. Twenty-one patients did not receive IBD treatment despite endoscopic signs of inflammation. Forty-one patients (65%) received a chemoprophylaxis and remission maintenance treatment with 5-aminosalycilate (mesalazin or sulfalazin, 5-ASA). Immunosuppressive treatment was administered in 17 patients (32%): 7 (10.9%) received azathioprin, 2 (3.1%) infliximab, an immunosuppressive combination therapy was given in 5 (7.8%) and cyclosporine in one patient (1.6%), one patients took budesonide (1.6%).

Twenty-three patients (35.9%) received chemotherapy with 5-Fu or doublet chemotherapy of 5-Fu combined with oxaliplatin or irinotecan. Systemic immunotherapy with pembrolizumab was administered in 2 patients with microsatellite instability.

Forty-seven patients with IBD-associated CRC were matched with patients with sporadic CRC. In both groups, 11 of 47 patients developed postoperative complications. With respect to Clavien-Dindo classification (CDC), the distribution of the severity of complications did not differ significantly (*p* = 0.633) - CDC grade I: 4 patients in each group; CDC grade II: 3 patients in each group; CDC grade III: 3 IBD patients and 2 sporadic CRC patients; CDC grade IV: 1 IBD patient. However, 2 patients of the sporadic CRC group died postoperatively (CDC grade V).

The median follow-up of all 3773 patients was 83 months (range 0–304); the median follow-up of the 1548 patients still alive was 145 months (range 40–304). For the 63 CED patients, the median follow-up was 36 months (range 1–228); for UC patients, 36 (range 1–216); and for CD patients, 24 months (range 3–228).

**Fig. 1:**
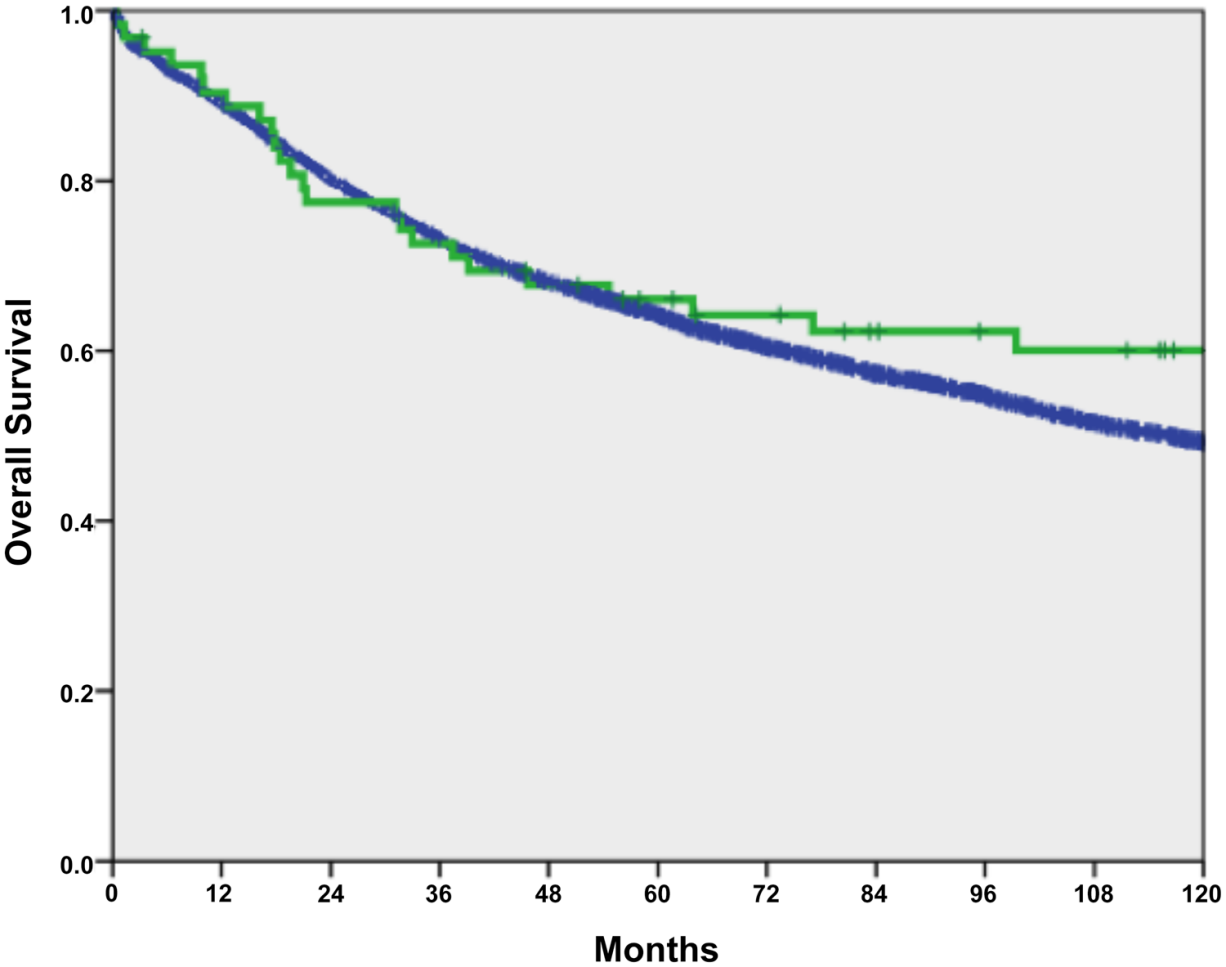
Overall survival between IBD with CRC (*n* = 63, green line) versus sporadic CRC (*n* = 3710, blue line), *p* = 0.270

**Fig. 2:**
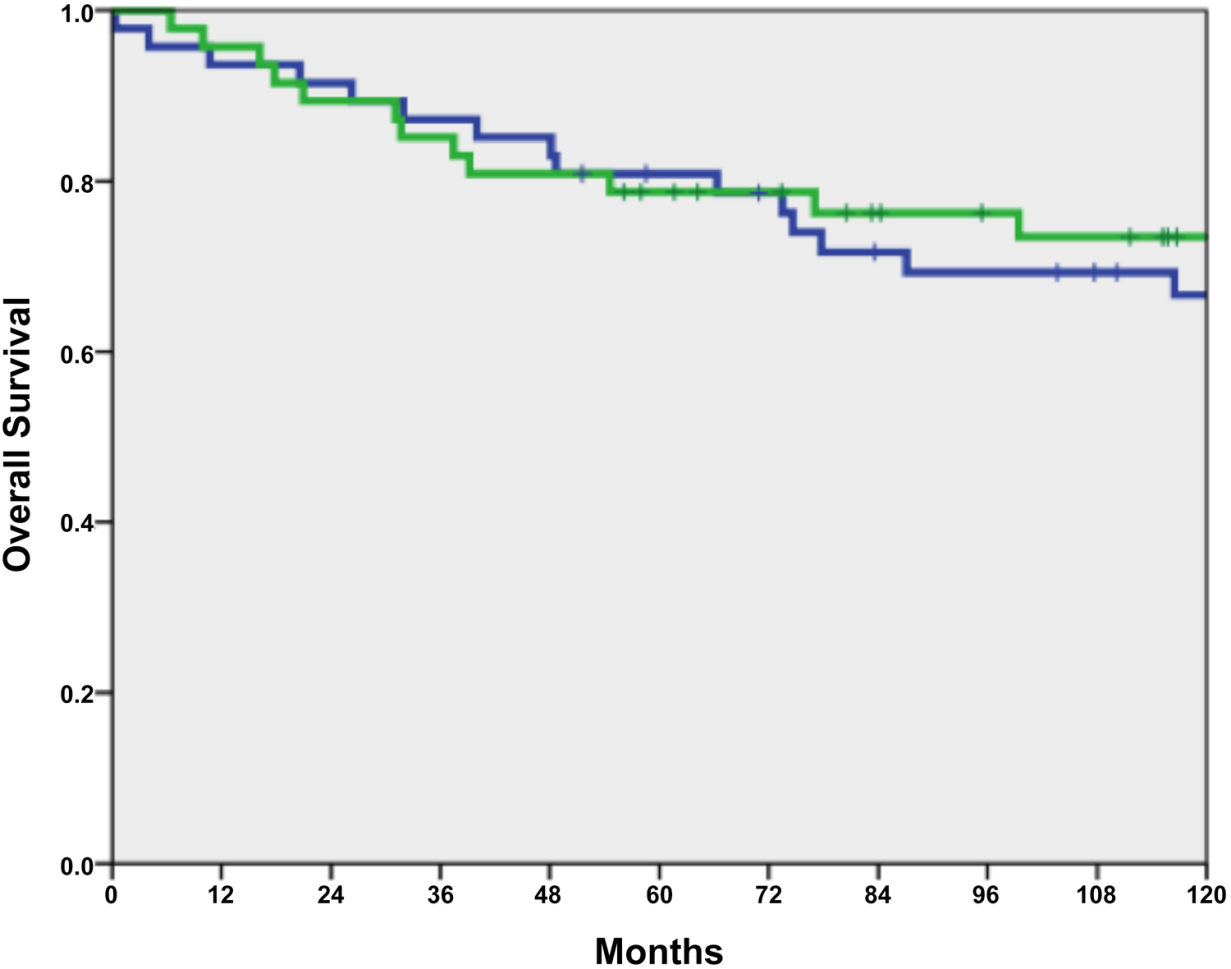
Overall survival of IBD-associated CRC (*n* = 47, green line) versus sporadic CRC (*n* = 47, blue line) in the matched-pair analysis, *p* = 0.990

**Fig. 3:**
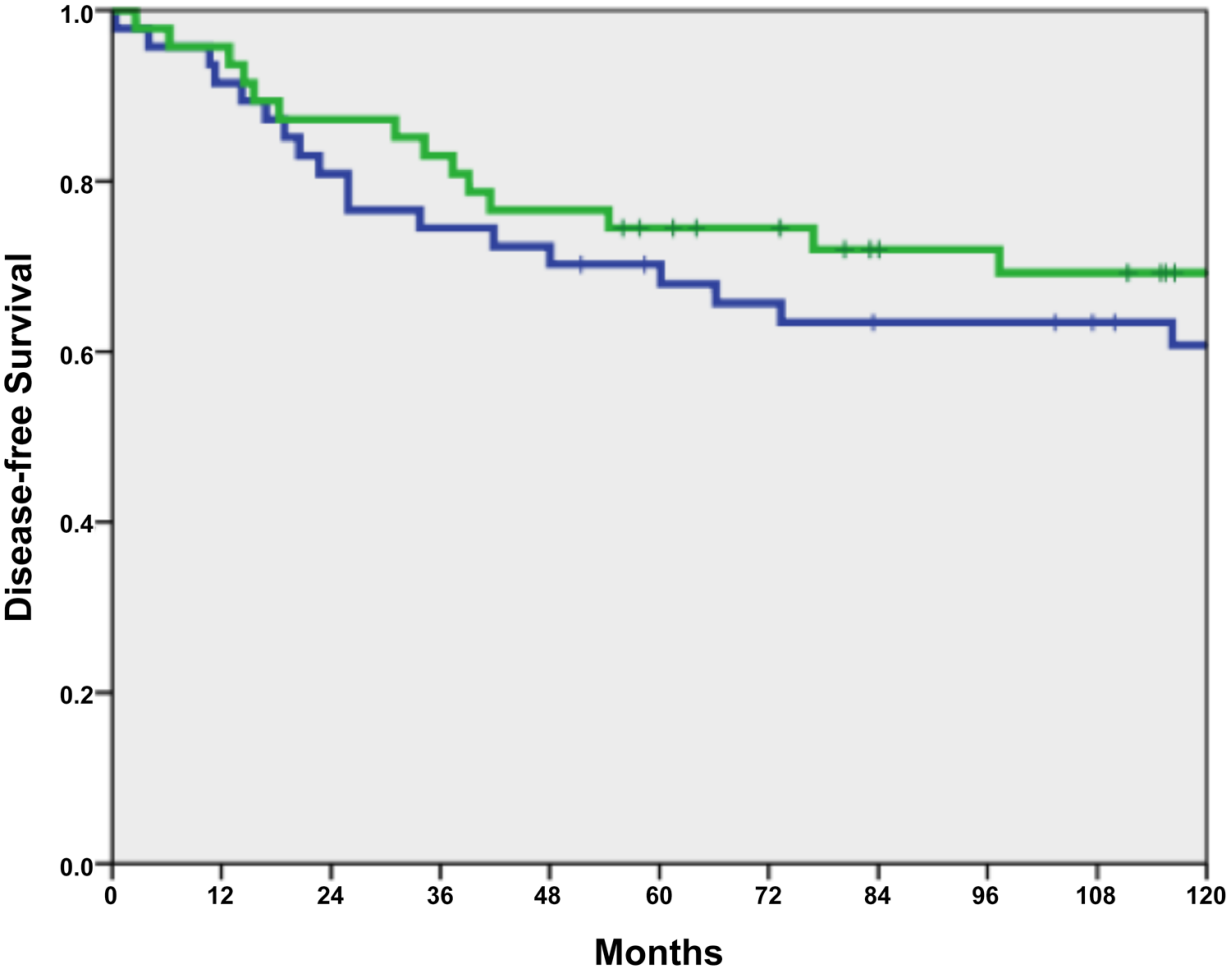
Disease-free survival of IBD-associated CRC (*n* = 47, green line) versus sporadic CRC (*n* = 47, blue line) in the matched-pair analysis, *p* = 0.593

Overall survival did not differ between 63 IBD-associated CRC patients and 3710 sporadic CRC patients (*p* = 0.270; Fig. 1). After matching 47 IBD-affected CRC patients with sporadic CRC patients, no differences in overall survival (OS) and disease-free survival (DFS) were found (*p* = 0.99 and *p* = 0.59; Table 3; Figs. 2 and 3).

**Table 3:** Overall and disease-free survival (*n* = 2 × 47)

	*n*	3-year rate (95% CI)	5-year rate (95% CI)	*p*
OS				
IBD-associated CRC	47	85.1 (74.9–95.3)	78.7 (66.9–90.5)	0.990
Sporadic CRC	47	87.2 (77.6/96.8)	80.9 (69.7–92.1)	
DFS				
IBD-associated CRC	47	83.0 (72.2–93.8)	74.5 (62.0–87.0)	0.593
Sporadic CRC	47	74.5 (62.0–87.0)	70.2 (57.1–83.3)

Of the 47 patients with IBD-associated CRC, a total of 9 patients developed distant metastases, 7 of them within 5 years. The 5-year rate of distant metastasis was 17.5%. The situation was similar in the 47 patients with sporadic CRC; 10 patients were diagnosed with distant metastases, 8 within 5 years; the 5-year rate of distant metastasis was 18.5% (*p* = 0.768). At the initial diagnosis of distant metastases, 6 IBD-associated CRC patients had metastases in a single organ and three patients had metastases in multiple organs. Similarly, sporadic CRC patients had solitary metastasis in 8 cases and multiple metastasis in 2 patients. The localization of the metastases was similar in both groups: liver in 2 patients each, lung in 3 and 6 patients, peritoneal metastases in 4 and 2 patients, others in 3 and 2 patients.

There were two local recurrences among IBD-associated rectal neoplasm (2/19; 11%) and one among sporadic rectal carcinomas (1/19; 5%; *p* = 0.57).

## Discussion

**Fig. 4:**
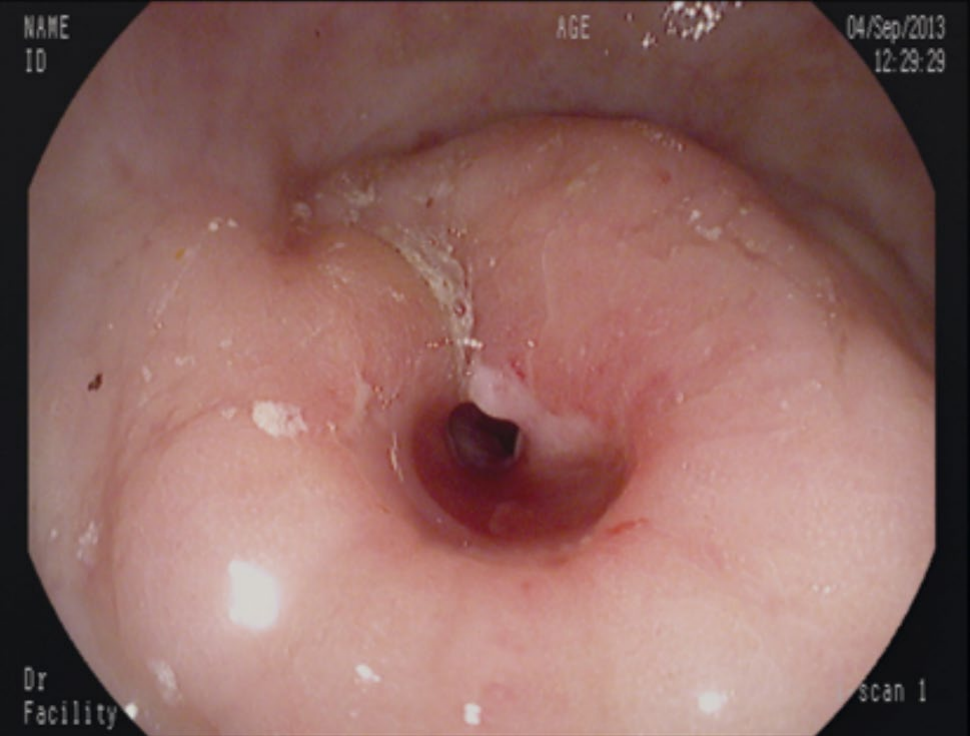
IBD-associated CRC in a patient presenting with a colonic stenosis

**Fig. 5:**
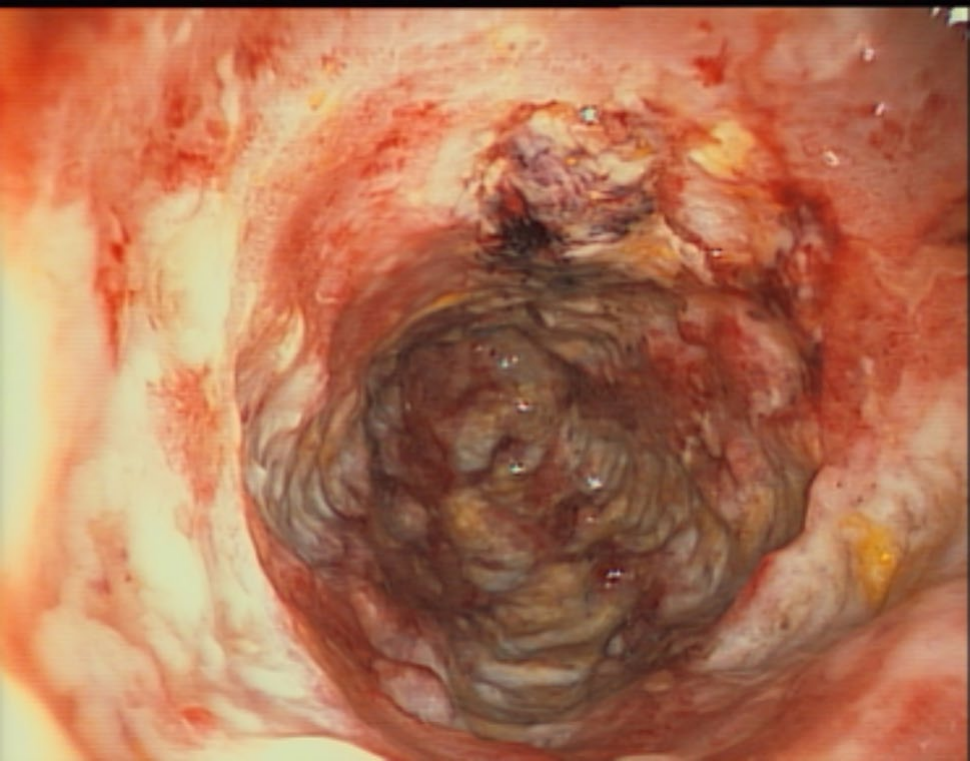
IBD-associated CRC with macroscopic signs of active inflammation. In the biopsies, a CRC was diagnosed

Over the last 30 years, the incidence of CRC in IBD has declined [15] and recent data estimate a cumulative CRC incidence for IBD of 1% at 10 years, 3% at 20 years, 7% at 30 years [3, 37]. This may reflect the impact of surveillance strategies, better control of inflammation with maintenance therapy, and increased resection rates for DALM or dysplasia. Endoscopy is a milestone technique for surveillance in the care of patients with IBD and in the prevention of CRC. In our cohort, 85.9% of the patients underwent surveillance. Although recommended [2, 38], none of the patients underwent dye-based chromoendoscopy; only surveillance based on random biopsies was performed during the study period. Even though surveillance was performed within the intervals recommended in the guidelines [1], CRC was diagnosed within a mean of 1.45 years after last endoscopy. The presence of cancer even in patients without overt signs of inflammation or malignancy on colonoscopy suggests the importance of histological evaluation and random biopsies in the surveillance of IBD patients, because fibrous tissue, pseudopolyps, and active inflammation can sometimes mask CRC (Figs. 4 and 5). According to such findings, we think that CRC screening should be improved and more effort should be done in applying cancer screening techniques like dye-based chromoendoscopy [39], artificial intelligence, and modern endoscopic imaging techniques [40].

In our cohort, CRC was diagnosed in patients with long-standing ulcerative colitis even in absence of endoscopically apparent inflammation. Such a finding was not emphasized in previous studies, but may suggest that inflammation patterns may trigger cancerogenesis in a complex and not yet completely understood way. Thus, colonic tissue may be at metachronous cancer risk even when inflammation can only be detected histologically [41]. Moreover, although a recently published retrospective survey reported that pseudopolyps do not increase the risk of malignancy [42], we found incidental CRC in biopsies from pseudopolyps of IBD patients without signs of severe inflammation. Pseudopolyps are probably not precancerous lesions per se, but are an indicator of past active relevant inflammation. Therefore, long-term follow-up (i.e., more than 5 years) should always be warranted in patients presenting with pseudopolyps.

In the literature, CRC has been shown to be diagnosed at an earlier stage in IBD patients, but survival is still worse compared to sporadic CRC and even the local recurrence risk was higher [43–46]. In our study, after excluding patients with distant metastasis and matching IBD-associated CRC with sporadic CRC, we found no difference in survival in patients after curative (R0) resection. Such findings underscore the importance of surveillance screening, because early diagnosis of a non-metastatic disease is associated with a prognosis comparable to that of sporadic CRC.

Usually CRC in IBD is diagnosed after about 10 years of disease duration; however, some data report early occurrence of CRC in IBD patients even after about 8 years [47]. In our study, 7 patients (7.9%) were diagnosed with CRC within 8 years since the diagnosis of IBD. This early incidence may be due to early onset of IBD and tobacco abuse (as risk of early CRC) [48]; however, we could not confirm such finding in our cohort. Such data suggest that surveillance should also be tailored also according to risk factors, not only duration of disease.

Histological findings in patients with IBD-associated CRC differed from sporadic CRC. IBD patients were diagnosed with rarer histological subtypes of CRC such as mucinous adenocarcinoma, signet ring cell carcinoma, adenosquamous carcinoma, medullary carcinoma, and undifferentiated carcinoma, in 19% compared to 9.2% in sporadic CRC. These histologic findings may explain a more aggressive behavior compared to the control CRC population, once metastases occur.

In this study, we did not compare survival between CD and UC patients, because such data have already been included in a recent analysis of our registry database [49].

For 47 patients with IBD that were matched with sporadic CRC (Table 4), no statistically significant difference in disease-free or overall survival was detected. The newly published data of the English National Cancer Registry database show that IBD patients tend to have a worse survival compared with sporadic cases of CRC, in particular for stage III disease with regional lymph node metastasis, while no difference between stages I and II was demonstrated [50].

**Table 4:** Patient characteristics of 94 (2 * 47) matched patients with colorectal carcinoma (match criteria in bold)

	IBD-associated CRC (*n* = 47)	Sporadic CRC (*n* = 47)	*p*-value
	*n* (%)	*n* (%)	
**Age**			
Median (range) (years)	46 (22–80)	51 (21–79)	0.166
**Sex**			
Male	30 (64)	30 (64)	1.0
Female	17 (36)	17 (36)	
ECOG*			
ECOG 0–1	40 (91)	38 (86)	0.739
ECOG 2–4	4 (9)	6 (14)	
ASA**			
ASA 1–2	35 (88)	39 (91	0.732
ASA 3–4	5 (12)	4 (9)	
**Tumor site**			
Right colon	18 (38)	18 (38)	1.0
Left colon	10 (21)	10 (21)	
Rectum	19 (40)	19 (40)	
**Emergency surgery**			
Yes	2 (4)	8 (17)	0.091
No	45 (96)	39 (83)	
**Surgery**			
TME/CME	23 (49)	47 (100)	< 0.001
(Procto)colectomy	24 (51)	0 (0)	
**Histological type**			
Adenocarcinoma (8140/3)	41 (87)	44 (94)	
Other histological types***	6 (13)	3 (6)	0.486
**Pathological stage (UICC)**			
Stage I	16 (34)	16 (34)	1.0
Stage II	16 (34)	16 (34)	
Stage III	12 (26)	12 (26)	
Stage yII	2 (4)	2 (4)	
Stage yIII	1 (2)	1 (2)	
**Multimodal treatment**			
Yes	16 (34)	13 (28)	0.509
NO	31 (66)	34 (72)	
Neoadjuvant treatment for rectal cancer	3/19	3/19	1.0
Adjuvant treatment	15/47	11/47	0.356
Adjuvant treatment for rectal carcinoma stages II and III	4/6	5/6	1.0
Adjuvant treatment for colon carcinoma stage III	6/7	5/7	1.0

*IBD* inflammatory bowel disease, *CRC* colorectal carcinoma, *ASA* American Society of Anesthesiologists Classification, *TME* total mesorectal excision, *CME* complete mesorectal excision. *ECOG performance status in 8 patients unknown; **ASA missing in 11 patients; ***undifferentiated carcinoma, mucinous adenocarcinoma, signet ring cell carcinoma

A limitation of this study is the retrospective nature of the study design and the limited number of patients included when compared to national cancer registries [10, 17, 50, 51]. Another drawback is the inability for proper age matching of sporadic CRC with IBD-associated CRC. However, since sporadic CRC is extremely rare and sometimes associated with an unusual genetic oncogenic mutation at young onset, this inability for age matching is directly reflecting different tumor biology.

The strength of the study is that we could evaluate endoscopic findings in the majority of the cases (more than 85% underwent surveillance endoscopy) and thus establish the severity of IBD inflammation, duration of disease, and intensity or type of immunosuppression.

In conclusion, we confirmed previous studies that IBD-associated CRC occurs at younger ages. However, in our study population, we did not prove worse survival in comparison to sporadic CRC in a score-matched analysis after excluding patients with metastatic disease. The results emphasize the importance of surveillance and early detection of CRC in patients with IBD. This should primary be performed by surveillance endoscopy with targeted biopsies in time intervals that should be adjusted to risk factors, particularly with a family history for CRC, PSC, long-standing disease and smoking [1, 8, 11]. New surveillance techniques such as virtual chromoendoscopy, artificial intelligence, and cellular imaging should be further developed in order to optimize CRC screening [39, 40], as advanced CRC occurs also in patients who underwent correct guideline-based endoscopic surveillance.
